# Investigation into the Anomalous Temperature Characteristics of InGaN Double Quantum Well Blue Laser Diodes Using Numerical Simulation

**DOI:** 10.1186/s11671-017-2141-6

**Published:** 2017-05-19

**Authors:** Han-Youl Ryu

**Affiliations:** Department of Physics, Inha University, Incheon, 402-751 South Korea

**Keywords:** Characteristic temperature, GaN, Quantum well, Laser diode, Carrier transport

## Abstract

GaN-based blue laser diodes (LDs) may exhibit anomalous temperature characteristics such as a very high characteristic temperature (*T*_0_) or even negative *T*_0_. In this work, temperature-dependent characteristics of GaN-based blue LDs with InGaN double quantum well (QW) structures were investigated using numerical simulations. The temperature-dependent threshold current is found to become increasingly anomalous as the thickness or doping concentration of the barrier layer between QWs increases. For a properly chosen barrier thickness and doping concentration, very high *T*_0_ of >10,000 K can be obtained. The anomalous temperature characteristics of these InGaN blue LDs are attributed to the increase of gain at the n-side QW with increasing temperature because of the thermally enhanced hole transport from the p-side to the n-side QW.

## Background

Recently, GaN-based blue laser diodes (LDs) have attracted great attention for use in laser-based white lighting [1–3] and visible light communication applications [4–7]. The blue LD-based white light source can eliminate the “efficiency droop” problem, which has been commonly observed in GaN-based light-emitting diodes (LEDs) at high injection currents [1, 8–10]. In LDs, the carrier density in the active region is clamped at the lasing threshold, and the injected carriers above threshold are converted to photons by stimulated emission, without undergoing nonradiative recombination. This leads to increased power conversion efficiency with increasing injection current in blue LDs. Therefore, LD-based white light sources can be advantageous for high-power and high-brightness illumination.

For high-power lighting applications, thermally stable operation of blue LDs is highly desirable. The temperature dependence of LDs is generally described by the following empirical expression [11]:

1 $$ {I}_{\mathrm{th}}={I}_0 \exp \left( T/{T}_0\right), $$

where *I*_th_ and *T* are the threshold current and absolute temperature, respectively. *I*_0_ is a constant having a dimension of current. *T*_0_ is the characteristic temperature, a phenomenological parameter that represents the temperature dependence of LDs. High *T*_0_ implies that *I*_th_ of LDs is less sensitive to the temperature change. If *I*_th_ is constant as temperature varies, *T*_0_ will go to infinity.

Normally, the *I*_th_ of LDs increases with *T* because of various temperature-dependent mechanisms such as thermal broadening of the gain spectrum, thermally activated carrier escape, free-carrier absorption, and Auger recombination. Most of the reported *T*_0_ values of GaN-based blue-violet LDs ranged from 120 to 180 K [12–15]. In some reports, however, anomalously high *T*_0_ of >200 K or even negative *T*_0_ values have been demonstrated [16–20]. Negative *T*_0_ implies that *I*_th_ decreases with increasing temperature. Bojarska et al. reported that the *T*_0_ of InGaN LDs increases as the emission wavelength increases owing to a reduction in the thermal escape of electrons from quantum wells (QWs) with increasing well depth [19]. Recently, they also observed negative *T*_0_ from a blue LD sample having a large distance between the electron-blocking layer (EBL) and the QWs, which was attributed to the thermal improvement of carrier injection efficiency [20]. Ryu et al. observed very high *T*_0_ and negative *T*_0_ in blue LD samples having double QWs [17, 18], which was ascribed to the thermally enhanced hole redistribution between the QWs. As the temperature increases, the carrier distribution in QWs become homogeneous, which is believed to increase gain in QWs and consequently decrease the lasing threshold. However, despite the experimental evidence of such anomalous temperature characteristics, thus far, the fundamental mechanism governing the value of *T*_0_ has not been clearly identified.

Numerical simulations of LD device characteristics may give insight into the origin of the anomalous *T*_0_ of InGaN blue LDs. In this paper, the temperature dependence of light output power as a function of injection current (*L*-*I*) in blue LD structures is numerically investigated using the simulation software, LASer Technology Integrated Program (LASTIP) [21]. LASTIP has been widely used in the study of semiconductor laser characteristics. In the present simulations, the active region is considered to be composed of InGaN double QW layers separated by a barrier layer. Since carrier transport and distribution in QWs is strongly influenced by the barrier layer between the QWs [18], the temperature dependence of the *L*-*I* curves is investigated as the thickness and doping concentration of the barrier layer are varied. In the next section, the simulated LD structure and the simulation method are described. In the “Results and Discussion” section, simulation results of various temperature characteristics are presented and the origin of the anomalous temperature characteristics in the InGaN blue LDs is discussed.

## Methods

**Fig. 1:**
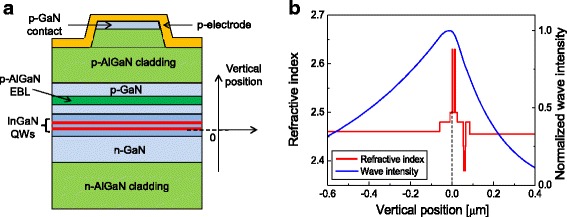
**a** Schematic of the simulated LD structure. **b** Vertical profiles of the refractive index (*left vertical axis*) and the normalized wave intensity of the lasing mode (*right vertical axis*) of the simulated LD structure

The LD structure for the simulation is basically similar to that reported in Ref. [18]. A schematic of the simulated LD structure is shown in Fig. 1a. The active region consists of two 2.5-nm In_0.15_Ga_0.85_N QW layers separated by an In_0.02_Ga_0.98_N barrier layer, which resulted in an emission wavelength of 449 nm. 1.2-μm n-Al_0.04_Ga_0.96_N and 0.6-μm p-Al_0.05_Ga_0.95_N layers are used as the bottom and the top cladding layer, respectively. A 20-nm p-Al_0.2_Ga_0.8_N EBL layer is employed to suppress electron leakage from the active region to p-GaN. A background electron concentration of 1 × 10^17^ cm^−3^ is assumed in the unintentionally doped QW and barrier layers [22]. Figure 1b shows profiles of the refractive index and the wave intensity of the lasing mode when the barrier thickness is 10 nm as a function of vertical position. The origin of the vertical position corresponds to the bottom interface of the n-side QW as shown in Fig. 1. The refractive index data of GaN, AlGaN, and InGaN alloys at 450 nm were adopted from Ref. [23]. The LD layer structures were designed so that the optical mode was asymmetrically distributed as shown in Fig. 1b. This design is known to be advantageous in avoiding the catastrophic optical damage of the laser mirror facet [14, 24]. The optical confinement factor of the lasing mode was calculated to be 0.63%. The LD chip structure has the form of a narrow-stripe ridge waveguide with a ridge width of 2.6 μm and a cavity length of 650 μm. Power reflectivity on the front and rear facet is 56 and 95%, respectively.

LASTIP self-consistently solves QW band structures, radiative and nonradiative carrier recombination, the drift and diffusion equation of carriers, and photon rate equations. The built-in electric fields induced by spontaneous and piezoelectric polarizations at the hetero-interfaces, InGaN/GaN and AlGaN/GaN, are also included using the model described in Ref. [25], assuming 50% compensation of the polarization fields [26, 27]. The conduction band offset of In_0.15_Ga_0.85_N/In_0.02_Ga_0.98_N active layers and AlGaN/GaN layers is set at 0.7 [10]. The Mg doping concentration of the p-GaN and p-AlGaN layers is fixed at 1 × 10^19^ cm^−3^, and the Mg acceptor ionization energy is assumed to scale linearly from 170 meV (GaN) to 470 meV (AlN) [10, 28]. In the n-type doped layer, the electron concentration is assumed to be the same as the doping concentration. The mobility model in Refs. [29–31] was used for the mobility of carriers, which gave an electron mobility of ~500 cm^2^/Vs for n-GaN with a doping concentration of 1 × 10^18^ cm^−3^. The hole mobility in the AlGaN, InGaN, and GaN layers is assumed to be 5 cm^2^/Vs [30].

**Fig. 2:**
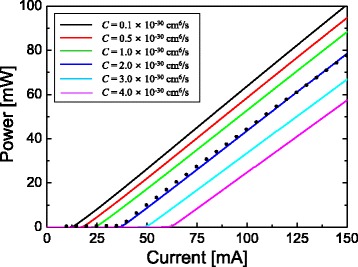
*L*-*I* curves of the simulated LD structures for various Auger recombination coefficient (*C*) values from 1 × 10^−31^ to 4 × 10^−30^ cm^6^/s at 20 °C. Here, the barrier thickness and doping concentration are fixed at 10 nm and 1 × 10^17^ cm^−3^, respectively. The *L*-*I* curve of the reference LD is represented as *solid dots*

In the carrier recombination model of LASTIP, the radiative recombination rate is calculated by integrating the spontaneous emission spectrum with a Lorentzian line-shape function. The Shockley-Read-Hall (SRH) recombination lifetime is assumed to be 20 ns. However, the effect of the SRH recombination on *I*_th_ was found to be almost negligible compared to the radiative or Auger recombination, if the SRH recombination lifetime is >10 ns. The *L*-*I* curve was found to depend strongly on the Auger recombination and the internal optical loss. The Auger recombination coefficient *C* and the internal loss *η*_*i*_ are chosen so that the simulated *L*-*I* curve can agree well with the measured one in Ref. [18]. The slope efficiency of the *L*-*I* curve is mainly determined by *η*_*i*_. When *η*_*i*_ is 10 cm^−1^, the slope efficiency is obtained to be ~0.7 W/A which corresponds fairly well to the measured slope efficiency of the reference LD structure in Ref. [18]. The internal loss of 10 cm^−1^ is also similar to that reported in InGaN blue LD structures [32, 33]. Figure 2 shows *L*-*I* curves for various *C* values from 1 × 10^−31^ to 4 × 10^−30^ cm^6^/s at 20 °C when the barrier thickness is set at 10 nm and the barrier doping concentration is 1 × 10^17^ cm^−3^. The *L*-*I* curve of the reference LD is plotted as solid dots [18]. As the coefficient *C* increases, *I*_th_ increases significantly, implying a strong influence of the Auger recombination on lasing threshold. When *C* is 2 × 10^−30^ cm^6^/s, the simulated *L*-*I* curve agrees quite well with the measured *L*-*I* curve of the reference LD. Therefore, in the simulation of this work, *C* and *η*_*i*_ are fixed at 2 × 10^−30^ cm^6^/s and 10 cm^−1^, respectively.

## Results and Discussion

**Fig. 3:**
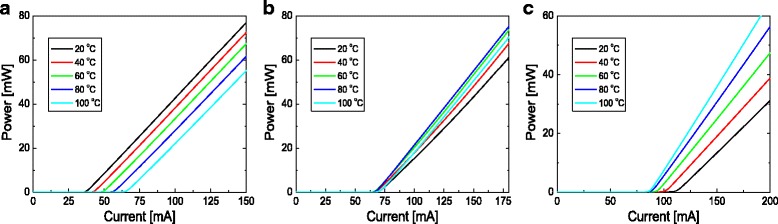
*L*-*I* curves for temperatures from 20 to 100 °C when the doping concentrations of the barrier are **a** 1 × 10^17^ cm^−3^, **b** 2 × 10^18^ cm^−3^, and **c** 3 × 10^18^ cm^−3^. Here, the barrier thickness is fixed at 10 nm

**Fig. 4:**
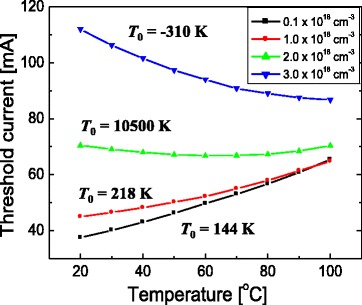
Threshold current (*I*_th_) as a function of temperature for barrier doping concentrations of 0.1, 1, 2, and 3 × 10^18^ cm^−3^. The *T*_0_ value for each doping concentration is shown. Here, the barrier thickness is fixed at 10 nm

In Fig. 3, the temperature dependences of *L*-*I* curves for different n-type doping concentrations of the barrier are compared when the barrier thickness is 10 nm. Figure 3a–c show simulated *L*-*I* curves for temperatures from 20 to 100 °C when the doping concentration of the barrier is 1 × 10^17^, 2 × 10^18^, and 3 × 10^18^ cm^−3^, respectively. Figure 4 plots *I*_th_ as a function of temperature for barrier doping concentrations of 0.1, 1, 2, and 3 × 10^18^ cm^−3^. When the doping concentration is 1 × 10^17^ cm^−3^, a normal temperature dependence of the *L*-*I* curves is observed. *I*_th_ increases steadily with temperature, resulting in a *T*_0_ value of 144 K. For the doping concentrations of 2 and 3 × 10^18^ cm^−3^, however, the temperature dependence of the *L*-*I* curves becomes anomalous. When the doping concentration is 2 × 10^18^ cm^−3^, *I*_th_ is almost unchanged with increasing temperature, resulting in a very high *T*_0_ of ~10,500 K. When the doping concentration is 3 × 10^18^ cm^−3^, *I*_th_ decreases with temperature, therefore, a negative *T*_0_ is obtained. This result indicates that the temperature dependence of *I*_th_ is strongly influenced by the doping concentration of the barrier layer in the InGaN blue LD structures. Figure 3b, c shows that the slope efficiency increases as the temperature increases, which has also been experimentally observed from a sample exhibiting negative *T*_0_ in Ref. [20]. The reason for this increase will be discussed later. It should be noted that the anomalous temperature characteristics, very high or negative *T*_0_, are accompanied by a substantial increase in the threshold current, which has also been observed experimentally [18, 20].

**Fig. 5:**
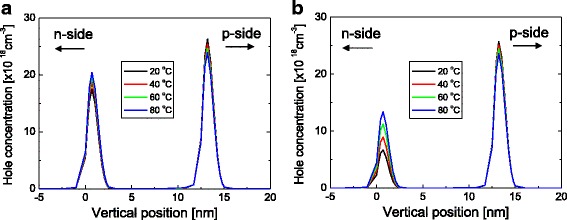
Hole concentration distribution at 30 mA for temperatures of 20, 40, 60, and 80 °C when the n-type doping concentration of the barrier are **a** 1 × 10^17^ cm^−3^ and **b** 2 × 10^18^ cm^−3^. Here, the barrier thickness is fixed at 10 nm

In order to understand the underlying mechanism related to the anomalous temperature characteristics, hole concentration and gain distribution in the QWs are calculated. Figure 5 shows the hole concentration distribution at 30 mA for temperatures of 20, 40, 60, and 80 °C when the n-type doping concentration of the barrier are (a) 1 × 10^17^ and (b) 2 × 10^18^ cm^−3^. For the low doping case (1 × 10^17^ cm^−3^), the hole concentrations of the two QWs are at a similar level and the hole distribution does not change significantly with temperature. For the high doping case (2 × 10^18^ cm^−3^), however, the hole concentration of the n-side QW is much lower than that of the p-side one. This is because the n-type doping effectively increases the potential barrier height for holes, preventing the holes from transporting from the p-side to the n-side QW. As the temperature increases, the hole concentration at the n-side QW increases as a result of thermally enhanced hole carrier transport from the p-side to the n-side QW. Consequently, hole distribution in QWs becomes increasingly homogeneous as temperature increases. In comparison to the temperature-dependent hole concentration, it was found that the electron distribution at two QWs did not change significantly with temperature because of the much higher mobility of electrons.

**Fig. 6:**
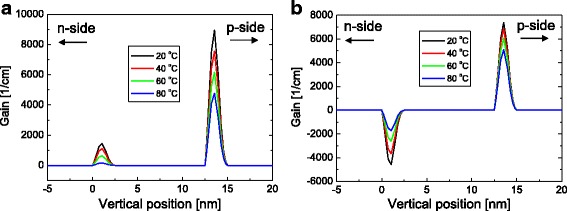
Gain concentration distribution at 30 mA for temperatures of 20, 40, 60, and 80 °C when the n-type doping concentrations of the barrier are **a** 1 × 10^17^ cm^−3^ and **b** 2 × 10^18^ cm^−3^. Here, the barrier thickness is fixed at 10 nm

Figure 6 shows the gain distribution below threshold at 30 mA for temperatures from 20 to 80 °C when the n-type doping concentration of the barrier are (a) 1 × 10^17^ and (b) 2 × 10^18^ cm^−3^. For both doping concentrations, gain of the p-side QW is larger than that of the n-side QW, because of the higher carrier concentration at the p-side QW. For the low doping case (1 × 10^17^ cm^−3^), gain at both QWs decreases as temperature increases. That is, the total gain decreases with increasing temperature mainly because of the thermal broadening of the gain spectrum [11]. As a result, *I*_th_ increased with temperature and normal temperature characteristics with *T*_0_ of 144 K were obtained as shown in Fig. 3a. For the high doping case (2 × 10^18^ cm^−3^), as temperature increases, gain at the n-side QW increases while gain at the p-side QW decreases. At the n-side QW, the increase of gain with increasing temperature results from the increase of the hole concentration with temperature as shown in Fig. 5b. In this case, the total gain changes only slightly with temperature, resulting in very high *T*_0_ of >10,000 K, as shown in Fig. 3b. It should be noted that the gain at the n-side QW was negative over the entire temperature range. That is, the n-side QW absorbs light instead of amplifying it, which results in the increase of *I*_th_ and the decrease of slope efficiency. Since the absorption at the n-side QW decreases with temperature, the slope efficiency increases as the temperature increases, as observed in Fig. 3b.

When the barrier doping concentration was 3 × 10^18^ cm^−3^, the total gain was found to increase with increasing temperature, resulting in the negative *T*_0_. The simulation results of hole and gain distribution in Figs. 5 and 6 imply that the anomalous temperature characteristics (very high or negative *T*_0_) of InGaN blue LDs are basically attributed to the thermal improvement of the hole injection efficiency from the p-side to the n-side QW layers.

**Fig. 7:**
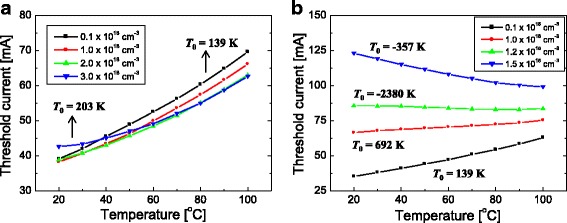
Threshold current (*I*_th_) as a function of temperature for barrier doping concentrations of 0.1, 1, 2, and 3 × 10^18^ cm^−3^ when the barrier thicknesses are **a** 6 and **b** 15 nm. The *T*_0_ value for each doping concentration is shown. Here, the barrier doping concentration is fixed at 1 × 10^18^ cm^−3^

Temperature-dependent characteristics are also investigated for other barrier thicknesses of 6 and 15 nm. Figure 7 plots the temperature dependence of *I*_th_ for a barrier thickness of (a) 6 and (b) 15 nm. When the barrier thickness is 6 nm, the temperature dependence of *I*_th_ is similar to that of when the doping concentration is from 1 × 10^17^ to 3 × 10^18^ cm^−3^ as shown in Fig. 7a. *T*_0_ increases from 139 to 203 K as the doping concentration increases from 1 × 10^17^ to 3 × 10^18^ cm^−3^. That is, the temperature dependence is basically normal for the entire simulation range of the doping concentration. This result implies that holes can transport efficiently from the p-side to the n-side QW even when the n-type doping concentration of the barrier is high if the barrier thickness is sufficiently thin.

When the barrier thickness is 15 nm, the temperature dependence of *I*_th_ becomes increasingly anomalous as the doping concentration increases. With this barrier thickness, the dependence of *T*_0_ on doping concentration is more anomalous compared to that of when the barrier thickness is 10 nm. For the low doping case (1 × 10^17^ cm^−3^), *T*_0_ is almost the same for all three-barrier thicknesses: 6, 10, and 15 nm. However, when the doping concentration is increased to 1 × 10^18^ cm^−3^, a high *T*_0_ of 692 K is obtained. When the doping concentration is higher than 1.2 × 10^18^ cm^−3^, a negative *T*_0_ can be obtained. This result implies that holes have difficulty in transporting from the p-side to the n-side QW when the barrier is relatively thick.

**Fig. 8:**
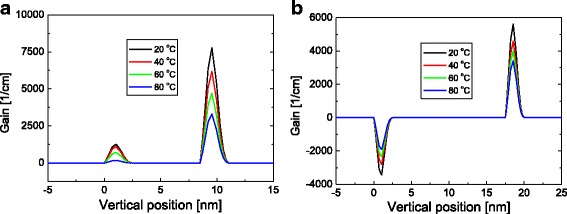
Gain concentration distribution at 30 mA for temperatures of 20, 40, 60, and 80 °C when the barrier thicknesses are **a** 6 and **b** 15 nm. The *T*_0_ value for each doping concentration is shown. Here, barrier doping concentration is fixed at 1 × 10^18^ cm^−3^

Figure 8 shows temperature-dependent gain distribution at 30 mA for the barrier thicknesses of (a) 6 and (b) 15 nm when the doping concentration is 1 × 10^18^ cm^−3^. When the thickness of the barrier is 6 nm, gain at both QWs is positive and decreases as temperature increases, which is similar to the result shown in Fig. 6a, where the barrier thickness is 10 nm and the doping concentration is 1 × 10^17^ cm^−3^. When the barrier thickness is 15 nm, gain at the n-side QW is negative and increases with temperature, as similarly observed in Fig. 6b, where the barrier thickness is 10 nm and the doping concentration is 2 × 10^18^ cm^−3^. This result implies that hole and gain distribution becomes increasingly inhomogeneous as the barrier thickness increases for a given doping concentration of the barrier. Consequently, the temperature characteristics become anomalous as the barrier thickness or doping concentration increases.

The anomalous temperature characteristics, very high or negative *T*_0_, may be advantageous for some applications that require thermally stable LD operation. However, such anomalous temperature characteristics are often accompanied by a substantial increase in *I*_th_ and deterioration of device efficiency. However, with careful design of the active layer structure, InGaN blue LDs with high efficiency and temperature-stable operation are expected to be realized for potential use in LD-based solid-state lighting, display, and communication applications.

## Conclusions

In this work, the temperature dependence of *I*_th_ in InGaN double QW blue LD structures was numerically investigated using the LASTIP simulator. Temperature dependent *I*_th_ and *T*_0_ were found to depend strongly on the n-type doping concentration and the thickness of the barrier layer between QWs. *T*_0_ was found to become increasingly anomalous as the thickness or doping concentration of the barrier layer between QWs was increased. When the barrier thickness was 10 nm and the doping concentration was 2 × 10^18^ cm^−3^, a very high *T*_0_ of >10,000 K was obtained. For a thicker barrier with higher doping concentration, negative *T*_0_ was observed. From the simulation of carrier and gain distribution in the QWs, it was found that the anomalous temperature characteristics originate from the increase in the gain at the n-side QW with increasing temperature as a result of the thermal enhancement of the hole transport from the p-side to the n-side QW. The result of this work is also expected to be applied to designing AlGaAs- or InGaAsP-based LD structures for thermally stable operation.

## Abbreviations

EBL: Electron-blocking layer
LASTIP: LASer Technology Integrated Program
LD: Laser diode
LED: Light-emitting diode
QW: Quantum well
SRH: Shockley-Read-Hall
